# Artificial Intelligence in Coronary Plaque Characterization: Clinical Implications, Evidence Gaps, and Future Directions

**DOI:** 10.3390/jcm15020903

**Published:** 2026-01-22

**Authors:** Juthipong Benjanuwattra, Cristian Castillo-Rodriguez, Mahmoud Abdelnabi, Ramzi Ibrahim, Hoang Nhat Pham, Girish Pathangey, Mohamed Allam, Kwan Lee, Balaji Tamarappoo, Clinton Jokerst, Chadi Ayoub, Reza Arsanjani

**Affiliations:** 1 Division of Cardiology, University of Cincinnati Medical Center, Cincinnati, OH 45219, USA; benjanjg@ucmail.uc.edu; 2 Department of Internal Medicine, Texas Tech University Health Sciences Center, Lubbock, TX 79430, USA; cristian.castillo-rodriguez@ttuhsc.edu; 3 Department of Cardiovascular Medicine, Mayo Clinic, Phoenix, AZ 85054, USA; ibrahim.ramzi@mayo.edu (R.I.); pathangey.girish@mayo.edu (G.P.); allam.mohamed@mayo.edu (M.A.); lee.kwan@mayo.edu (K.L.); tamarappoo.balaji@mayo.edu (B.T.); ayoub.chadi@mayo.edu (C.A.); arsanjani.reza@mayo.edu (R.A.); 4 Department of Medicine, University of Arizona, Tucson, AZ 85719, USA; npham917@arizona.edu; 5 Department of Cardiovascular Medicine, Mayo Clinic, Rochester, MN 55905, USA; 6 Department of Radiology, Mayo Clinic, Phoenix, AZ 85054, USA; jokerst.clinton@mayo.edu

**Keywords:** artificial intelligence, coronary artery disease, coronary artery calcium, coronary computed tomography angiography, atherosclerosis, coronary plaque, coronary plaque quantification, plaque characteristics

## Abstract

Coronary artery disease (CAD) remains the leading cause of cardiovascular morbidity and mortality worldwide, with plaque composition and morphology being as key determinants of disease progression and clinical outcomes. Accurate plaque characterization is essential for risk stratification and therapeutic decision-making, yet conventional image interpretation is limited by inter-observer variability and time-intensive workflows. Artificial intelligence (AI) models have emerged as a transformative tool for automated coronary plaque analysis across multiple imaging modalities. AI-driven models demonstrate high diagnostic accuracy for plaque detection, segmentation, quantification, and vulnerability assessment. Integration of AI-derived imaging biomarkers with clinical risk scores can further enhance prediction of major adverse cardiovascular events and supports personalized management. These advances position AI-enhanced imaging as a powerful adjunct for both invasive and non-invasive evaluation of CAD. Despite its promise, important barriers to widespread clinical adoption remain, including data heterogeneity, algorithmic bias, limited model transparency, insufficient prospective validation, regulatory challenges, and incomplete integration into clinical workflows. Addressing these challenges will be essential to ensure safe, generalizable, and cost-effective implementation of AI in routine cardiovascular care.

## 1. Introduction

Coronary artery disease (CAD) remains a leading cause of cardiovascular morbidity and mortality worldwide. One of the key determinants of CAD outcomes is the characterization of coronary plaques, which can differentiate stable plaques from those prone to rupture with acute coronary syndrome. In the current era, artificial intelligence (AI) has emerged as a powerful tool in medical imaging, with significant applications in the analysis of coronary plaque in various cardiovascular diseases [1]. Through machine learning (ML) and deep learning (DL) algorithms, AI has shown promise in addressing limitations of manual interpretation of imaging data, which is traditionally time-consuming and requires expertise [1]. This review article discusses the current evidence on AI’s roles across various imaging modalities in automated coronary plaque detection and characterization, evidence gaps, and future directions (Table 1: Summary of the role of AI in different imaging modalities).

## 2. Role of AI in Coronary Imaging

AI applications in coronary plaque imaging include automated detection and segmentation of coronary vessels, precise quantification of plaque components, prediction of plaque vulnerability, and workflow automation across multiple imaging modalities. (DL) models—particularly convolutional neural networks (CNN) and U-Net-based models—achieve high agreement with expert readers for plaque and lumen segmentation while significantly reducing analysis time to seconds [2,3]. AI enables precise quantification of plaque components including calcified, fibrous, lipid-rich, and necrotic core tissue, showing strong correlation with invasive imaging standards [4]. AI-based plaque metrics demonstrate significant prognostic value, with higher total plaque volume and lipid-rich features associated with increased myocardial infarction risk [3]. Emerging multimodal models that integrate clinical parameters and multimodality imaging data aim to standardize analysis and support decision-making, while automating various imaging tasks such as coronary calcium scoring, functional flow reserve (FFR) estimation, and CAD-RADS classification [5].

## 3. Intravascular Ultrasound (IVUS)

Intravascular Ultrasound (IVUS) is a valuable imaging modality for in vivo assessment of coronary plaque morphology. It can guide percutaneous coronary intervention (PCI) from pre-interventional to post-interventional phases, including assessing lesion length, stent expansion, and minimal stent area [6]. By emitting ultrasound waves from a catheter-based transducer, IVUS provides high-resolution cross-sectional visualization of the vessel wall and lumen [7]. Based on grayscale echogenicity, plaques are classified as soft, fibrous, calcified, or mixed. The presence of a necrotic core—manifesting as attenuated or echolucent plaque—is frequently observed in culprit lesions and is associated with increased risk of peri-procedural myocardial infarction [7]. Multiple studies have demonstrated the superiority of IVUS-guided PCI over angiography-guided intervention [6]. Specific plaque features, including a thin fibrous cap, a large plaque burden, and a minimal luminal area ≤4.0 mm^2^, independently predict major adverse cardiac events (MACEs) [8]. Despite its advantages, IVUS remains limited by inter- and intra-observer variability and the time-intensive nature of manual frame-by-frame analysis. Plaque morphologies not only have prognostic value but can also support clinical decision-making in the treatment of patients with CAD. Previous studies showed that a thin fibrous cap, large plaque burden, and minimal luminal area of 4.0 mm^2^ or less independently predicted major adverse cardiac events (MACEs) [8]. Despite its advantages, IVUS remains limited by inter- and intra-observer variability and the time-intensive nature of manual frame-by-frame analysis [9]. AI-based automated segmentation of IVUS pullback images enables rapid, reproducible, and comprehensive quantification of vessels and plaques, supporting real-time clinical decision-making [10]. Early applications include IVUS-Net, which delineated the lumen and external elastic membrane in 0.15 s per frame [11], and U-Net, which accurately predicted minimal lumen area and severe calcification, though with limitations in stent detection [12]. EfficientNet further improved characterization of calcified and attenuated plaques with ≥93% accuracy and enabled precise quantification of lumen and plaque volumes [10,13]. More recently, DeepLabv3 demonstrated strong performance in simultaneous segmentation of lumen, vessel, and stent areas, achieving a mean intersection-over-union of 0.80 and processing 100 frames in 4 s [14]. Nevertheless, high-acoustic-impedance structures, such as stent struts and heavily calcified lesions, remain persistent technical challenges. Beyond segmentation, AI enables automated tracking of plaque progression over time, potentially allowing for serial monitoring of disease response to lipid-lowering therapies. Real-time AI analysis during PCI may also assist operators by flagging high-risk plaque features or suboptimal stent deployment, reducing procedural complications [15].

## 4. Optical Coherence Tomography (OCT)

Optical coherence tomography (OCT) is a high-resolution intravascular imaging modality that uses near-infrared light to generate cross-sectional images with superior spatial resolution compared with IVUS, albeit with limited tissue penetration [16]. Because it is unaffected by the high acoustic impedance of calcium, OCT is particularly useful for the assessment of calcific coronary lesions [16]. Although OCT offers faster pullback speeds than IVUS, image interpretation remains time-consuming and subject to inter- and intra-observer variability, requiring substantial expertise due to complex plaque morphology and frequent imaging artifacts. These limitations underscore the growing need for AI-based tools to enhance diagnostic accuracy and clinical efficiency. OCT is especially valuable for evaluating plaque vulnerability. Its high resolution enables precise measurement of fibrous cap thickness and identification of lipid-rich plaques, both of which are associated with an increased risk of future major adverse cardiac events (MACEs) [17]. In addition, OCT-guided rotational atherectomy-assisted PCI allows assessment of calcium cracks and arc thickness, parameters that predict optimal stent expansion in heavily calcified lesions [18]. Collectively, these capabilities support improved risk stratification and personalized treatment planning [17,19]. Recent studies highlight the expanding role of AI in OCT analysis. Min et al. demonstrated that a deep learning model accurately identified thin-cap fibroatheroma in 602 coronary lesions, achieving 92.8% frame-level and 91.3% pullback-level accuracy [20]. A DenseNet-121-based algorithm further showed that AI-detected vulnerable plaques were independently associated with higher MACEs compared with AI-classified stable plaques [19]. Another AI-assisted OCT study reported diagnostic accuracies of 97.6% for fibrous plaque, 90.5% for lipid plaque, and 88.5% for calcium plaque [21]. In real-world OCT-guided PCI, Katagiri et al. demonstrated that the convolutional neural network-based system “Ultreon” showed strong correlation with expert visual assessment of calcification angle, thickness, and length in 85% of lesions, despite a 13% false-positive rate primarily in acute coronary syndrome cases [22]. More recently, the automated platform OCTPlus has been applied for plaque characterization before and after PCI in patients receiving third-generation everolimus-eluting bioabsorbable magnesium scaffolds [23]. Overall, AI-enabled OCT analysis improves the speed and precision of plaque characterization and facilitates reliable identification of high-risk features—including thin fibrous caps and large lipid cores—that are closely linked to plaque rupture and adverse cardiovascular events.

## 5. Coronary Computed Tomography Angiography (CCTA)

Coronary computed tomography angiography (CCTA) is a noninvasive imaging modality that uses ionizing radiation and advanced scanners to generate high-resolution three-dimensional images of the coronary arteries, enabling both diagnosis and risk stratification to guide clinical management. Current guidelines provide a Class I, Level A recommendation for CCTA in patients with chest pain at intermediate–high risk of CAD [24]. Beyond assessment of luminal stenosis, CCTA offers comprehensive quantitative and qualitative evaluation of plaque burden and composition, which more accurately predicts future major adverse cardiovascular events (MACEs) [25]. However, diagnostic performance may be limited by heavy calcification, tachycardia, arrhythmias, and the presence of coronary stents [25]. Before the advent of AI, plaque quantification relied on manual interpretation, a process that is time-consuming and prone to interobserver variability. Early semi-automated approaches demonstrated clinical value, such as QFAT v2.0, which quantified pericoronary adipose tissue attenuation—a marker of coronary inflammation—to differentiate patients with acute myocardial infarction, stable CAD, and healthy controls [26]. More recently, Hu et al. introduced DeepFat, a deep learning algorithm applied to coronary calcium scans that quantifies epicardial adipose tissue morphology and attenuation, both of which strongly predict MACEs [27]. AI-driven quantitative CT (AI-QCT) has markedly improved reproducibility of plaque assessment. In the CLARIFY study, Jonas et al. demonstrated high interobserver agreement for plaque volume quantification (concordance correlation coefficient [CCC] = 0.95), as well as acceptable reproducibility for minimal lumen area (0.91), minimal lumen diameter (0.86), and plaque burden (0.80) [28]. Reproducibility declined with decreasing plaque density—ranging from CCC 0.98 for dense calcium to 0.84 for necrotic core—highlighting the importance of AI in reducing observer variability [23]. AI-QCT also correlated strongly with near-infrared spectroscopy–IVUS in quantifying plaque burden, lesion length, lumen area, and vessel area in lipid-rich noncalcified plaques, achieving sensitivity and specificity of 93% and 94%, respectively [29]. Data from the ADVANCE registry further reinforced the prognostic role of AI-enhanced CCTA. Abnormal FFRCT FFR (≤0.80) was independently associated with increased MACEs [30]. Using an automated deep learning model, AI-QCPA, Dundas et al. evaluated plaque volumes in 4430 patients and demonstrated that total plaque volume independently predicted MACEs even after adjustment for FFRCT [31]. The REVEALPLAQUE study confirmed strong agreement between AI-QCPA-based CCTA and IVUS for plaque quantification and characterization (total plaque volume, lumen volume, calcified plaque volume, and non-calcified plaque volume) in patients with stable CAD, though both modalities showed limitations in measuring low-attenuated plaque [32]. To standardize reporting, the Coronary Artery Disease Reporting and Data System (CAD-RADS) classification was developed to integrate stenosis severity, plaque burden, and modifiers including FFRCT and myocardial perfusion [33]. Multiple studies have shown that AI-automated CAD-RADS classification using deep learning closely correlates with expert interpretation [34,35,36]. Incorporation of AI-QCPA into the DECODE study resulted in substantial CAD-RADS reclassification, prompting intensification of medical therapy in many patients [37]. Additionally, Lin et al. applied a hierarchical convolutional long short-term memory (ConvLSTM) network to over 5045 lesions from the SCOT-HEART trial, demonstrating strong agreement with expert readers and IVUS for plaque volume, percent stenosis, and minimal lumen area, with total plaque volume independently associated with myocardial infarction [38]. Overall, these findings have proven the potential of AI-driven CCTA as an alternative to invasive IVUS for assessing plaque burden and characterization.

## 6. Cardiac Magnetic Resonance Imaging (MRI)

Cardiac magnetic resonance imaging (MRI) is the gold standard for quantifying cardiac structure and function offering unparalleled capability for detailed myocardial tissue characterization [39]. Additionally, it can noninvasively detect plaque components, including fibrous cap, calcification, and macrophage-rich lesions [40]. However, coronary magnetic resonance angiography requires longer acquisition times than CCTA and is particularly susceptible to motion-related blurring and artifacts due to the small caliber of coronary arteries and respiratory and cardiac motion, limiting its widespread clinical adoption despite its potential as a comprehensive “one-stop” imaging modality [41]. To overcome these limitations, several AI-integrated techniques have been developed to shorten acquisition time and improve image quality [39,42]. A study by Wu et al. demonstrated that compressed sensing with AI reconstruction reduced acquisition time by 22% while preserving high diagnostic image quality for the detection of CAD [38]. Similarly, Muñoz et al. integrated a self-supervised diffeomorphic non-rigid respiratory motion estimation network (DiRespME-net) into GPU-based motion-corrected iterative reconstruction, achieving total reconstruction times of approximately 20 s with preserved coronary vessel sharpness and length [43]. AI-based approaches for automated plaque characterization using MRI have also been validated in extracoronary settings, such as carotid artery stenosis [44], although further studies are needed to establish their reliability for coronary plaque segmentation.

## 7. Single Photon Emission Computed Tomography (SPECT)/Positron Emission Tomography (PET)

AI algorithms can be applied to myocardial perfusion imaging workflow using SPECT/PET, encompassing image acquisition, registration, reconstruction, segmentation, diagnosis, and risk stratification [45]. AI-assisted denoising and reconstruction techniques enable shorter scan times while potentially reducing radiation exposure without compromising image quality [45]. Although coronary artery calcium (CAC) scoring does not localize disease or determine hemodynamic significance, it provides important prognostic information and guides optimization of medical therapy. Deep learning-based automated calcium scoring, performed in less than one second, has demonstrated strong agreement with expert interpretation and robust prediction of future MACEs [46]. Moreover, integration of CT-derived calcium scores with stress PET imaging using machine learning models—such as the XGBoost algorithm—has achieved diagnostic accuracy comparable to expert readers for identifying obstructive CAD [47]. Kwiecinski et al. further applied the XGBoost algorithm to define the risk of myocardial infarction in patients with advanced CAD by combining quantitative coronary CT angiography plaque analysis with 18F-sodium fluoride PET [48]. Collectively, these advances highlight the expanding role of AI in enhancing the diagnostic and prognostic capabilities of PET and SPECT for comprehensive coronary plaque assessment.

## 8. AI Integration with Clinical Risk Scores

Hybrid approaches integrating AI with clinical risk scores, such as the Framingham risk score and CAC scoring, have shown promise in optimizing predictions for coronary plaque characterization and subsequent cardiovascular outcomes. Several studies have demonstrated the enhanced predictive power of combining AI with traditional clinical risk scores. For instance, Tesche et al. found that a machine learning (ML) model integrating coronary CT angiography (cCTA)-derived plaque measures and clinical parameters obtained significantly improved risk scores. Compared to conventional CT risk scores and clinical parameters alone, MACE prediction achieves an area under the curve (AUC) of 0.96 [49]. Similarly, Benjamins et al. reported that ML models integrating clinical and cCTA variables outperformed traditional methods in identifying patients with myocardial ischemia and those requiring early revascularization. The best performance was achieved by combining expert CTA interpretation with clinical variables (AUCs = 0.91 and 0.90). ML using all CTA variables achieved an AUC of 0.85, which was outperformed by expert CTA interpretation (AUC = 0.87). The best performance was achieved by ML integrating expert CTA interpretation and clinical variables, with AUCs of 0.91 and 0.90 for identifying myocardial ischemia and early revascularization, respectively [50]. Additionally, Nurmohamed et al. demonstrated that AI-guided quantitative plaque staging using coronary CT angiography (AI-QCT) provided significant prognostic value for long-term cardiovascular outcomes, improving the model’s performance over clinical risk factors and CAC scoring alone (10-year AUC: 0.82 vs. 0.73) [51]. Beyond traditional risk scores, emerging AI models incorporate multimodality imaging data, serial biomarker trends, and clinical data to improve risk predictions which can potentially individualize prevention strategies.

**Table 1:** Comparison of Imaging Modalities for AI-driven Coronary Plaque Characterization.

Modality	Resolution	AI Readiness	Invasiveness	Plaque Characterization Accuracy	Current Limitations	Representative AI Tool/Study
IVUS	Moderate (100–150 µm)	High—Multiple DL frameworks tested	Invasive	>90% (calcification, MLA, plaque burden)	Artifacts from stents/calcifications, interobserver variability	IVUS-Net, DeepLabv3 (Nishi et al.) [14]
OCT	High (~10–20 µm)	High—Commercial tools emerging	Invasive	Up to 97.6% (fibrous/lipid/calcified plaques)	Limited penetration, artifacts, complex interpretation	Ultreon AI, DenseNet-121 (Katagiri et al.) [22]
CCTA	Moderate (~300–600 µm)	Very High—Widely validated models	Non-invasive	CCC > 0.95 for total plaque volume	Radiation exposure, limited by artifacts as heavy calcifications or motion, or elevated HR	AI-QCT, AI-QCPA, DeepFat (Jonas et al. [28], Lin et al. [38])
Cardiac MRI	Moderate (~1 mm spatial)	Moderate—Early adoption phase	Non-invasive	Validated in carotid plaques, coronary pending	Long scan time, motion artifacts, and technical barriers	DiRespME-net, Compressed sensing AI (Wu et al. [38], Munoz et al. [43])
SPECT/PET	Low (~4–6 mm)	Moderate—Applied in denoising, fusion algorithms	Non-invasive	Strong correlation with CT and expert readers	Low spatial resolution, indirect indicators, no direct localization	XGBoost integration with PET, automated CAC scoring (Kwiecinski et al. [48])

IVUS: Intravascular ultrasound, OCT: Optical Coherence Tomography, CCTA: Coronary Computed Tomography Angiography, MRI: Magnetic Resonance Imaging, SPECT/PET: SPECT (Single Photon Emission Computed Tomography) and PET (Positron Emission Tomography), MLA: Minimal Lumen Area, HR: Heart Rate, CCC: Concordance Correlation Coefficient.

## 9. Challenges and Future Directions

Despite the potential of AI applications in coronary plaque characterization, their implementation in routine clinical practice faces several challenges:

- Data Quality and Standardization: AI algorithms require large, high-quality, and diverse datasets for training, which are often limited in availability and can significantly impact diagnostic accuracy, reliability, and generalizability when applied to diverse patient populations [52].
- Transparency and explainability of AI algorithms: Black-box AI models may provide predictions without clear explanations, posing clinical acceptance and reliability challenges. Additionally, the lack of clear recommendations for reporting how AI models are developed, tested, and validated—including details on sample size, patient demographics, and imaging equipment—makes assessing their usefulness in clinical settings harder [52].
- Algorithmic Bias: AI algorithms can have the same biases in the original training data, resulting in disparities in diagnostic performance in different clinical scenarios. Addressing these biases carefully is required to ensure adequate performance during model development and validation [5].
- Validation: Many AI systems lack prospective validation in real-world clinical settings. Multi-center trials are required to validate the safety and effectiveness of AI algorithms in coronary plaque characterization in different patient populations before widespread adoption [52].
- Integration in Clinical workflow: Challenges such as interoperability, data management, workflow integration, continuous validation, effective change management, and clear ethical and legal guidance should be addressed to ensure that AI models can be integrated into electronic health records (EHRs) and imaging platforms to be useful in practice [5,53].
- Cost-Effectiveness: Data regarding the cost-effectiveness of AI-assisted plaque characterization models are still lacking. Theoretically, they have significant potential for improving healthcare expenses; however, their cost-effectiveness, successful implementation, and generalizability depend on addressing specific local challenges related to infrastructure, data sharing, and training, which have yet to be determined [54].
- Regulatory Approval and Ethical Concerns: Before widespread utilization in routine clinical practice, AI tools must be comprehensively evaluated for their safety and efficacy while maintaining patient privacy and data security. Continuous post-market surveillance and transparent communication between developers and regulatory bodies are essential to maintaining the integrity and trustworthiness of AI tools in healthcare [55,56].

## 10. Conclusions

Emerging evidence suggests that AI-based plaque analysis models can improve plaque detection, quantification, and characterization and assist in risk stratification and clinical decision-making. However, limitations such as data standardization, model transparency, regulatory approval, and clinical integration should be addressed before widespread adoption. Further research is required, focusing on multicenter validation, interpretability, and real-world applications to determine the feasibility of AI integration in clinical practice.

## Data Availability

All data used in this review article are from published content. Please see the reference lists.
